# Novel Strategies in the Early Detection and Treatment of Endothelial Cell-Specific Mitochondrial Dysfunction in Coronary Artery Disease

**DOI:** 10.3390/antiox12071359

**Published:** 2023-06-28

**Authors:** Weiqian E. Lee, Elijah Genetzakis, Gemma A. Figtree

**Affiliations:** 1 Kolling Institute, University of Sydney, Sydney, NSW 2006, Australia; eugene.lee@sydney.edu.au (W.E.L.); elijah.genetzakis@sydney.edu.au (E.G.); 2 Faculty of Medicine and Health, University of Sydney, Sydney, NSW 2006, Australia; 3 Department of Cardiology, Royal North Shore Hospital, Northern Sydney Local Health District, Sydney, NSW 2065, Australia

**Keywords:** coronary artery disease, endothelial dysfunction, mitochondrial dysfunction, endothelial colony forming cells, drug screening, atherosclerosis, SMuRFless

## Abstract

Although elevated cholesterol and other recognised cardiovascular risk factors are important in the development of coronary artery disease (CAD) and heart attack, the susceptibility of humans to this fatal process is distinct from other animals. Mitochondrial dysfunction of cells in the arterial wall, particularly the endothelium, has been strongly implicated in the pathogenesis of CAD. In this manuscript, we review the established evidence and mechanisms in detail and explore the potential opportunities arising from analysing mitochondrial function in patient-derived cells such as endothelial colony-forming cells easily cultured from venous blood. We discuss how emerging technology and knowledge may allow us to measure mitochondrial dysfunction as a potential biomarker for diagnosis and risk management. We also discuss the “pros and cons” of animal models of atherosclerosis, and how patient-derived cell models may provide opportunities to develop novel therapies relevant for humans. Finally, we review several targets that potentially alleviate mitochondrial dysfunction working both via direct and indirect mechanisms and evaluate the effect of several classes of compounds in the cardiovascular context.

## 1. Introduction

Unmet needs in coronary artery disease—beyond traditional risk factors—modifications in lifestyle, and the use of pharmacotherapeutic agents such as statins, anti-hypertensives, and anti-thrombotic therapies have been at the forefront of preventing, managing, and treating coronary artery disease (CAD) in at-risk individuals [1,2]. However, CAD remains the leading cause of mortality and morbidity worldwide, representing 32% of total deaths reported in 2019 [3]. In addition, there is an increasing presence of myocardial infarction (MI) in the absence of standard modifiable risk factors (SMuRFs), which highlights the urgent need to unravel novel biosignatures and mechanisms in CAD [4,5]. A notable group that represents up to 27% of first-presentation ST-elevation myocardial infarction (STEMI) patients is termed ‘SMuRF-less’ and was demonstrated to suffer a 47% higher 30-day mortality compared to those who presented with at least one standard modifiable risk factor [6,7]. Moreover, findings from both studies elucidate the insufficiency of our current biological understanding of CAD development beyond the ‘lipid-driven’ narrative that has determined an individual’s susceptibility to atherosclerosis.

Current approaches for diagnosing CAD susceptibility and disease are through two anatomic investigations, either computer tomography coronary angiography (CTCA) or coronary artery calcium scoring (CACS) [8,9]. Screening with either of these measures in patients with low traditional risk factor measures is not recommended by international guidelines on cardiovascular disease prevention and risk [4,5]. As such, this poses a major obstacle in diagnosing those with subclinical CAD. Therefore, discovering a biomarker capable of identifying subclinical CAD that could be confirmed by non-invasive CT imaging is the holy grail of the cardiovascular field. Using such markers to identify individuals from low-risk groups with CAD would allow prevention therapy to stabilise existing plaque, thereby halting plaque progression and, consequently, heart attack. Thus, this posits the need for not only new markers to facilitate novel strategies to detect CAD but also innovations in screening that could be translated to a clinical setting. 

Recently, mitochondrial dysfunction from endothelial cells has been shown to correlate with CAD burden and is a strong candidate for the development of scalable assays for clinical application. Therefore, targeting mitochondrial dysfunction represents an untapped potential in the detection and treatment of CAD. This review will examine the mitochondria and their impact on causing atherosclerosis, targets, and potential compounds to treat mitochondrial dysfunction as well as future directions in this field. 

## 2. Mitochondrial Dysfunction as an Instigator of Endothelial Dysfunction in Coronary Artery Disease

### 2.1. Mitochondria: Oxidative Phosphorylation and the Production of Mitochondria ROS 

**Figure 1:**
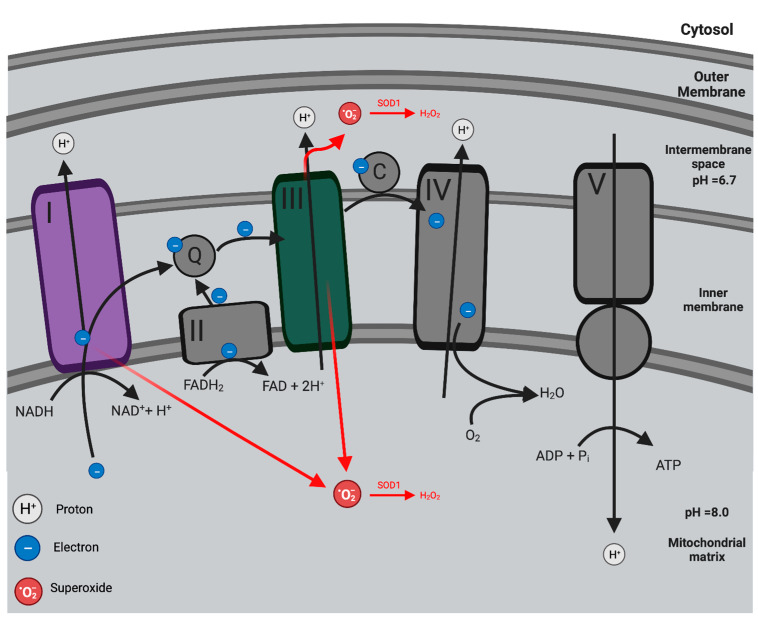
Oxidative phosphorylation and mitochondrial ROS (mROS) production. Nutrient oxidation in the TCA cycle releases free energy as reduced cofactors NADH and FADH_2_. Electrons from NADH and FADH_2_ are transported across Complexes I–IV, forming the ETC. Through a series of redox reactions, the transport of electrons is coupled with the translocation of protons (H^+^) across the inner mitochondrial membrane and into the intermembrane space. This process generates the proton motive force (PMF), driving ATP synthesis as H^+^ re-enters the mitochondrial matrix through Complex V (ATP synthase). Thus, nutrient oxidation is coupled with ATP production. mROS are produced from the leakage of e- at Complex I and III to form superoxide (O_2_^•−^). O_2_^•−^ is generated in the mitochondrial matrix at Complex I while O_2_^•−^ is released to both the mitochondrial matrix and intermembrane space at Complex III. Once formed, O_2_^•−^ undergoes dismutation by either SOD1 (in the intermembrane) or SOD2 (in the mitochondrial matrix) into H_2_O_2_. Created with BioRender.com (accessed on 7 June 2023).

The chemiosmotic theory postulated by Peter Mitchell in 1961 explained the relationship between ATP production and the electron transport chain (ETC) [10]. Electrons are generated by the tricarboxylic acid (TCA) cycle when nutrients and metabolic intermediates are broken down during oxidative reactions [11]. These electrons are transferred and held by the reduced cofactors NADH and FADH_2_ [12]. Electrons are then shuttled from these reduced cofactors via Complexes I–IV of the electron transport chain in a series of redox reactions that terminate with the reduction of O_2_ to H_2_O [13]. The ETC drives proton pumps in the inner mitochondrial membrane that efflux protons from the matrix to create an electrochemical proton gradient or proton motive force (PMF) [10]. This electrochemical potential generated from the PMF is harnessed to synthesise ATP [12]. ADP from the mitochondrial matrix is phosphorylated to become ATP when protons re-enter the mitochondrial matrix via the F_1_F_0_ ATP synthase (Complex V) [12]. Overall, oxidative phosphorylation involves the coupling of nutrient oxidation and electron transport to ATP production (Figure 1).

However, the highly reduced ETC leaks electrons, enabling the generation of mitochondria ROS (mROS) [14]. Electron leakage primarily occurs at Complex I and III (and, to a lesser extent, Complex II), leading to the partial reduction of oxygen, thereby forming superoxide (O_2_^•−^) [15]. Three leakage events occur within this pathway: O_2_^•−^ is released from Complex I towards the mitochondrial matrix, while Complex III leaks O_2_^−^ towards both the mitochondrial matrix and the intermembrane space [16,17]. Afterward, O_2_^•−^ is dismutated into H_2_O_2_ by either superoxide dismutase 1 (SOD1) in the intermembrane space or superoxide dismutase 2 (SOD2) in the mitochondrial matrix (Figure 1) [15].

### 2.2. Pathogenesis of Mitochondrial Dysfunction in Endothelial Dysfunction 

The mitochondria are known as the powerhouse of the cell, notable for playing key roles in ATP production through oxidative phosphorylation, calcium homeostasis, and innate immunity regulation [18,19]. However, they have gained increasing interest due to their primary role in ROS production [20]. They produce O_2_^•−^ which subsequently undergoes dismutation into hydrogen peroxide (H_2_O_2_) [20]. Endothelial mitochondria are highly sensitive environmental damage sensors that play a prominent role in signalling cellular responses when exposed to mechanical or biochemical stressors, as experienced in hypertension, hypercholesterolemia, and diabetes mellitus [21]. When exposed chronically to noxious stimuli, the ETC complexes in the endothelial mitochondria become damaged, leading to eventual dysfunction and excessive production of mROS [22]. 

Excessive mROS are pro-inflammatory and cause oxidative damage, further damaging the cell and the mitochondria and creating a self-perpetuating, vicious cycle of inflammation and oxidative stress [23]. In this context, ROS are important signalling molecules that activate pro-thrombotic and pro-inflammatory pathways in the endothelium, resulting in the transformation from endothelial dysfunction to the eventual development of atherosclerotic plaques [24]. 

The cause of endothelial mitochondrial dysfunction stems from a variety of factors. Typically it is characterised by redox imbalance and vascular tone dysregulation, which contributes to increased inflammatory responses within the blood vessel wall [25,26]. Exposure to stimuli such as LDL activates the molecular machinery of the endothelium, leading to the expression of chemokines, cytokines, and adhesion molecules which then interact with leukocytes and platelets in response to injury, and it ultimately results in reactive oxygen species (ROS) production [27]. ROS are formed by cells as by-products of cellular metabolism and act as important intermediary messengers within cells, transducing intracellular signals involved in biological processes [28]. However, chronic exposure to superoxide and its by-products can exhaust the anti-inflammatory system of endothelial cells and lead to an imbalance [28]. These processes lead to oxidative stress as elevated levels of ROS cause cell injury, thus promoting the progression of several diseases ranging from cancer to cardiovascular disorders [29,30].

Initially, excessive mROS is managed several ways. Superoxide dismutases such as manganese superoxide dismutase (MnSOD) convert O_2_^•−^ in the mitochondrial matrix into H_2_O_2_, which is later broken down by antioxidant enzymes, like catalase and peroxidase, into water and oxygen [31]. Another measure that also protects against mROS are other mitochondrial proteins, such as paraoxonase 2 (PON2) and uncoupling proteins (UCPs), which inhibit mROS production [32,33,34,35]. However, if mROS production exceeds the buffering capacity of the mitochondria’s antioxidant defense system, oxidative and cellular damage ensues. O_2_^•−^ is highly reactive and can damage various components of the mitochondria, including proteins, lipids, and mitochondrial DNA (mtDNA) as well as ETC protein complexes, with damage to the latter increasing mROS production, initiating a positive feedback loop and exacerbating endothelial dysfunction and vascular disease [31]. 

With the mitochondria identified as a major source of cellular superoxide as well as being associated with endothelial dysfunction in early atherosclerosis, it has great potential as a biomarker for the early detection and characterization of CAD. The potential of endothelial mitochondria as a significant target has previously been vastly overlooked. This is mainly due to vascular endothelial cells predominantly utilising anaerobic glycolysis for ATP turnover as well as the mitochondria only comprising 2–6% of endothelial cell volume [21,36,37,38]. When compared to other cell types with high energy requirements, such as hepatocytes (28%) and cardiomyocytes (32%), this is relatively small and may indicate that oxidative phosphorylation through the mitochondria is not important [21,31,38]. However, it is now recognised that oxidative phosphorylation plays a critical role in cell signalling through ROS generation and its role in calcium homeostasis [39]. In addition, endothelial mitochondria have been shown to switch to oxidative phosphorylation for ATP production in order to meet increased metabolic demand through the concept known as ‘mitochondrial reserve capacity’ [40]. 

### 2.3. Mitochondrial Dysfunction in Atherosclerosis

**Figure 2:**
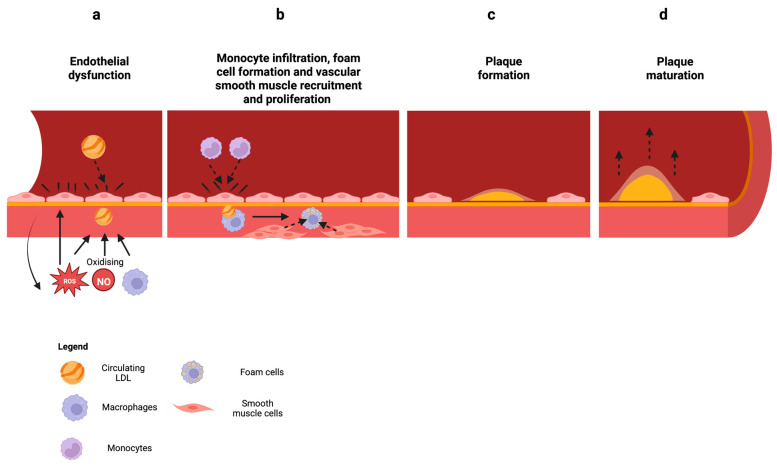
Progression of atherosclerosis. (**a**) Atherogenic factors such as elevated and modified low-density lipoprotein (LDL) and reactive oxygen species (ROS) cause endothelial dysfunction. Circulating LDL accumulates in the intima of coronary vessels and is oxidised by factors such as ROS, nitric oxide (NO), and macrophages. ROS also directly damages the endothelium and creates a perpetuating cycle. (**b**) Oxidized LDL activates the endothelium, attracting monocytes to the intima that differentiate into macrophages. Oxidized LDL is phagocytosed by macrophages, becoming foam cells that later form fatty streaks. Fatty streaks attract smooth muscle cells (SMCs) to the lesion site and produce extracellular matrix. (**c**) The atherosclerotic plaque then forms through the accumulation of large amounts of extracellular matrix, leading to the thickening of the arterial intima. (**d**) The plaque forms a fibrous cap around the lipid core, which builds up and narrows the arteries, eventually leading to complications or myocardial infarction (MI). Created with BioRender.com (accessed on 7 June 2023).

Oxidative stress and endothelial dysfunction have long been associated with inflammatory diseases like CAD. In particular, endothelial dysfunction is well-recognised as an early predictor of atherosclerosis and is profoundly associated with cardiovascular disorder pathogenesis [25,41,42,43,44]. Atherosclerosis occurs when there is endothelial dysfunction caused by atherogenic factors such as elevated and modified low-density lipoprotein (LDL) and ROS [45,46] (Figure 2a). LDL accumulates in the intima of the coronary vessels whereby they undergo oxidation through exposure to nitric oxide, macrophages, and enzymes such as lipoxygenase [47,48]. ROS is also able to oxidize lipids through lipid peroxidation, which leads to the formation of hydroperoxidised lipids and an alkyl radical [49]. These oxidised LDLs promote the activation of the endothelium, attracting monocytes into the intima that later differentiate into macrophages [50,51] (Figure 2b). Oxidised LDLs are scavenged and phagocytosed by macrophages which become foam cells (another hallmark of atherosclerosis) and form fatty streaks [52]. These fatty streaks trigger signals which attract smooth muscle cells (SMCs) to the lesion where they proliferate and produce an extracellular matrix consisting of collagen and proteoglycans [53]. As a result, the atherosclerotic plaque develops, which leads to the thickening of the arterial intima and the formation of a fibrous cap around the lipid core [46] (Figure 2c,d). This plaque build-up causes the narrowing of arterial vessels, reducing blood flow to the heart and other parts of the body. A complication of CAD is myocardial infarction (MI), or heart attack, caused by plaque build-up in the coronary arteries, which become unstable and rupture. This leads to the formation of blood clots which can obstruct blood flow to the heart [54]. 

Despite this, neither measurement nor treatment of these risk factors in humans has yet been achieved. Hence, tackling the current limitations in measuring dysregulated redox signalling may provide new avenues to detect individual susceptibility. As such, models of human cellular redox stress which reflect atherosclerotic disease processes would be beneficial for the development of new treatments. 

## 3. Mitochondrial Dysfunction as a Biomarker in the Early Detection of Coronary Artery Disease

### 3.1. Targeting Mitochondria ROS and Oxidative Stress

The rationale behind using therapies that target the mitochondria lies in the key roles that it plays in regulating energy synthesis, ROS production, and apoptosis. In order to target mROS, we must understand the three strategies to target mitochondria pharmacology [55]. The first is by targeting compounds to the mitochondria which can be achieved by the conjugation to a lipophilic cation like TPP or Szeto-Schiller (SS)/mitochondrial-processing peptidase (MPP) peptides such that the compound can be transported to the mitochondria matrix by the selective uptake of the attached bioactive moiety or pharmacophore [56,57]. Another strategy is to use compounds that do not selectively target the mitochondria but can act upon them when binding to specific targets [58]. Lastly, therapeutic agents that can modulate or influence mitochondrial dysfunction by affecting processes outside the mitochondria, such as kinase activity, transcription factors, and transcriptional coactivators should be considered [55].

In addition, we can measure the effectiveness of these compounds through in-vitro and in-vivo assays that measure mitochondrial superoxide production. Dikalov and Harrison have previously outlined several methods of detecting mitochondrial superoxide production in depth [59]. In particular, the probe MitoSOX has been used extensively to measure mitochondrial superoxide production in vitro. Watanabe et al. found that the mean fluorescence intensity of MitoSOX in CAD-positive, monocyte-derived macrophages was almost double compared to healthy patients [60]. Another paper by Owada et al. utilised MitoSOX to measure the effect of MitoTempo, a mitochondria-specific antioxidant, on alleviating mitochondria oxidative stress in the cardiomyocytes of aged mice [61]. They were able to confirm differences between ROS production in aged and young mice as well as demonstrate MitoTempo’s usefulness in scavenging mitochondrial superoxide [61]. Recently, another mitochondria-targeted superoxide probe, MitoNeoD, has been developed for in vivo and in vitro usage. MitoNeoD was capable of detection in mice when injected with rapid uptake [62]. When comparing wild-type mice and mice administered MitoParaquat (MitoPQ), a compound that selectively increases mitochondrial superoxide production in the heart, MitoNeoD detected approximately 1.5 times higher signal in MitoPQ-treated mice [62,63]. 

Mitochondrial dysfunction can also be assessed through mitochondrial respiration. One method is high-resolution respirometry (HRR), which utilizes air-tight reaction chambers and oxygen sensors with high sensitivity and precision to provide accurate amperometric measurements of oxygen consumption rate (OCR). HRR was performed in the hearts of Zucker obese fatty (ZOF) rats, revealing a 33% decrease in state-3 respiration and a 50% decrease in maximal oxygen consumption compared to Zucker lean (ZLN) rats. [64]. In a study involving humans, the utilization of HRR revealed that mitochondrial respiration in the arterial fibers of patients with coronary heart disease (CHD) and diabetes was inhibited in both the NADH-linked and succinate-linked pathways [65]. This inhibition resulted in an overall decrease in mitochondrial respiratory rates compared to controls without CHD [65,66]. 

Other methods have also been developed to detect biomarkers of mitochondrial dysfunction. An example is utilising and investigating metabolomics data to detect metabolic signatures for diagnosis and potential treatment targets. This was performed to identify novel circulating biomarkers of mitochondrial dysfunction in patients with heart failure (HF) and preserved ejection fraction (HFpEF) versus those with reduced ejection fraction (HFrEF) [67]. A fully adjusted model comparing metabolite factors between HFpEF, HFrEF, and healthy patients was able to individually uncover 6 long-chain acylcarnitine (LCAC) metabolites that were differentially significant between the three groups [67]. These results identify a specific metabolic pathway in heart failure (HF), indicating that the application of metabolomics in large-scale clinical studies holds significant promise for identifying novel pathways associated with CAD. Translation of these findings should be evaluated in combination with current tools to unveil potential biosignatures in early CAD patients. 

### 3.2. Potential Targets to Reduce Mitochondrial ROS Production and Oxidative Stress

#### 3.2.1. Mitochondria Outer Membrane Proteins 

Proteins of the mitochondria outer membrane include porins and voltage-dependent anion channels (VDACs), small pore-forming proteins found in all eukaryotic cells [68]. VDAC is important for regulating apoptosis through the interaction with proteins of the Bcl-2 family [69]. VDAC has also been linked to protecting against oxidative stress, where downregulation or absence of VDAC has been shown in numerous studies to increase the production of ROS [70,71,72]. VDAC3 has also been shown to counteract ROS-induced oxidative stress [73]. 

#### 3.2.2. Mitochondria Inner Membrane Proteins 

The mitochondrial inner membrane proteins are part of the ETC that constitute oxidative phosphorylation [74]. As mentioned earlier, Complex I and III function as the primary sites for ROS generation (Complex II to a lesser extent), all due to electron “leakage”. This process ultimately results in the reduction of molecular oxygen [14]. Complex III has been shown to have several functions which make it an ideal therapeutic target [75]. It generates significant levels of ROS, controlling mitochondrial calcium influx, which regulates mitochondrial permeability transition (MPT) and cell death as well as acting as an environmental oxygen sensor for hypoxia [75,76,77]. A study conducted by James et al. demonstrated that inhibiting Complex III resulted in significant disruptions in the mitochondrial proteome, affecting 28 proteins in rats with sustained pulmonary vasoconstriction induced by antimycin A [78]. Furthermore, the inhibition of Complex III significantly dysregulated mitochondrial fatty acid metabolism, the tricarboxylic acid (TCA) cycle, and the electron transport chain (ETC). Several proteins involved in fatty acid breakdown, as well as key enzymes in the TCA cycle, including aconitase (ACOC) and 2-oxoisovalerate dehydrogenase subunit beta (ODBB), were found to be significantly downregulated [78]. Therefore, compounds that either protect or upregulate Complex III can serve as protein targets to preserve mitochondrial function.

Other potential protein targets within the inner mitochondrial membrane include UCPs which dissipate the proton gradient without the formation of ATP having significant downstream effects on oxidative stress [79]. UCP2 is particularly noteworthy due to its ubiquitous expression in all cell types and its recognized role in protecting against oxidative stress in atherosclerosis and vascular dysfunction [80]. Overexpressing UCP2 in THP-1 monocytes has been shown to decrease intracellular ROS production in response to H_2_O_2_, TNF-α, and monocyte chemoattractant protein-1 (MCP-1) [81]. Additionally, in human aortic endothelial cells (HAECs) challenged with lysophosphatidylcholine (LPC) and linoleic acid, overexpression of UCP2 significantly increased state 3 and state 4 respiration [35]. Consequently, compounds capable of upregulating UCP2 expression would provide a beneficial protein target to safeguard against mitochondrial dysfunction.

Optic atrophy protein (OPA1) is another potential candidate, as its loss is associated with increased mitochondrial ROS production and mitochondrial dysfunction [82,83,84]. OPA1 plays multiple roles in remodeling and stabilizing the mitochondrial cristae, including the regulation of mitochondrial cytochrome *c* release and the improvement of mitochondrial respiratory efficiency by facilitating the assembly of electron transport super-complexes [83,84,85]. Previous research has explored the targeting of OPA1 as a cardioprotective strategy using epigallocatechin gallate (EGCG). In these studies, EGCG attenuated OPA1 cleavage in H_2_O_2_-treated mouse embryonic fibroblasts (MEFs), effectively protecting the cells from mitochondrial fission and fragmentation [86]. Consequently, EGCG may hold promise in treating mitochondrial dysfunction caused by atherosclerosis.

### 3.3. Mitochondria DNA

Mitochondrial DNA (mtDNA) defects contribute significantly to arterial hardening, where it is observed early in the pathogenesis of atherosclerosis in the walls of blood vessels and has been shown to cause pro-atherogenic factors such as inflammation and apoptosis [87,88]. Due to its proximity to the ETC, mtDNA is a vulnerable target of ROS and oxidative damage, lacking protective histone proteins, and poor repair mechanisms against damage [89]. It has also been reported that mtDNA is more susceptible to oxidative stress than nucleic DNA [87,90,91]. Damaged mtDNA also has the potential to amplify oxidative stress. For example, mutations in Complex I genes may lead to Complex I deficiency, resulting in an increase in mROS production [92,93]. Failure of DNA repair could lead to ketosis, hyperlipidemia, and increased storage of fat, which may promote atherosclerosis development [31]. As mitochondrial function is dependent on proteins that are encoded by nucleic and mitochondrial DNA, the latter is considered a preclinical marker of atherosclerosis and presents as a potential therapeutic target [94]. Approaches include repopulating cells with wild-type, unmutated mtDNA and/or the elimination or repair of mutated, dysfunctional mtDNA [95,96]. 

#### 3.3.1. P66^SHC^

P66^SHC^ is a mitochondrial protein that regulates mitochondria function and ROS production [97]. It is present in the mitochondrial intermembrane space where it functions as a redox enzyme, oxidizing cytochrome *c*, and partially reducing molecular O_2_ to H_2_O_2_ [98]. Studies involving p66^SHC^-knock out mice have been shown to be resistant to oxidative stress and cardiovascular pathologies, such as atherosclerosis and endothelial disorders [99,100]. In human umbilical vein endothelial cells (HUVECs), overexpression of p66^SHC^ was found to promote endothelial dysfunction, which included phenotypes, such as ROS generation, E-selection expression, and leukocyte transmigration [101]. Thus, targeting p66^SHC^ could be viable as a therapeutic target to combat mROS generation and restore endothelial function. 

#### 3.3.2. P2X7 Receptor

The P2X7 receptor (P2X7R) is a type of purinergic receptor that is expressed in endothelial cells and is involved in a variety of cellular functions, including the regulation of cellular inflammation and oxidative stress [102,103,104]. When activated, the P2X7 receptor has been shown to increase the production of mROS and oxidative stress in endothelial cells [105]. The increase in mROS production has been attributed to several mechanisms, which include mitochondrial permeabilization, NADPH oxidase activation, and decreased antioxidant defenses [31]. Activation of P2X7R can lead to mitochondrial permeabilization, which allows for the release of ROS from the mitochondria into the cytoplasm, subsequently leading to oxidative stress and cellular damage [106,107,108]. In addition, P2X7 receptor activation has been shown to affect the NADPH oxidase complex, which plays a key role in the production of ROS in cells [109,110]. Deletion of P2X7R resulted in decreased levels of antioxidant enzymes, such as superoxide dismutase (SOD) and catalase (CAT) which aid in scavenging ROS and preventing oxidative stress [111]. Mice with P2X7R deficiency (P2X7R^−/−^) were protected against mitochondrial stress measured by SOD/CAT activity ratios in a model of sepsis [111]. 

In the instance of atherosclerosis, turbulent blood at the lesion site causes a significant increase in ATP, a potent activator of the P2X7R, which leads to downstream signalling effects such as p38 activation which can lead to further inflammation, oxidative stress, and endothelial dysfunction [112,113,114]. Overall, P2X7R activation can contribute to the development of endothelial dysfunction and cardiovascular disease and serves as a potential therapeutic strategy for the treatment of cardiovascular disease and related disorders. 

### 3.4. Myeloperoxidase

Myeloperoxidase (MPO) is an enzyme that is primarily produced by neutrophils and monocytes and has been implicated in the development of endothelial dysfunction by limiting NO bioavailability through the production of hypochlorous acid (HOCl), a potent oxidant [115]. HOCl can cause oxidative damage to various cellular components including the mitochondria, causing mitochondrial and endothelial dysfunction [116,117]. La Rocca et al. demonstrated that endothelial cells produced MPO [118]. Primary cultures of human endocardial endothelial cells (EEC) from the hearts of patients with chronic heart failure (CHF) and HUVECs that were subjected to H_2_O_2_-induced oxidative stress (60 μM) led to MPO expression [118]. Elevated levels of MPO have been found in the blood and tissues of patients with these conditions and have been associated with an increased risk of adverse outcomes such as myocardial infarctions and strokes [119,120]. As such, MPO is a significant therapeutic candidate to alleviate oxidative stress, offering a promising solution to endothelial and mitochondrial dysfunction and atherosclerosis.

## 4. Potential Therapeutic Strategies to Target Mitochondrial ROS and Oxidative Stress

For translation to human therapy, pharmacotherapeutics to treat mitochondrial dysfunction in endothelial cells and prevent the accumulation of ROS should have low cytotoxicity, mitochondria-specific and/or superoxide scavenging activity, and an appropriate half-life for treatment in a cardiovascular/atherosclerotic setting. As shown in Table 1, the compounds and their classes have been appraised and found to fit some but not all of the criteria. 

### 4.1. Mitochondrial Uncouplers

Protons typically re-enter the matrix via Complex V through the coupling of nutrient oxidation and ATP production; however, they can also move into the matrix with the aid of carrier proteins or small molecule carriers [138]. Protons that re-enter the matrix without producing ATP ‘uncouple’ the ETC from ATP production [138]. This process is known as mitochondrial uncoupling or proton leak. Mitochondrial uncoupling is a natural process that occurs through both basal and inducible proton leaks [138]. In rat hepatocytes, basal proton leak contributes to approximately 20–30% of the basal metabolic rate (BMR), while in rat skeletal muscle, it can account for as much as 50% [14,139]. In addition, proton leaking can also be induced by specialised proteins such as UCPs and adenine nucleotide translocases (ANTs), which occur naturally [138,140].

Mild mitochondrial uncoupling limits the formation of ROS by decreasing the mitochondrial membrane potential (Δψm) generated by proton pumps (Complex I, III, and IV) as well as decreasing the local oxygen availability due to higher oxygen consumption [141,142]. This prevents the oversupply of electrons to the respiratory chain, which would minimise the probability of electron leaks, and thus decreases the production of O_2_^−^ in the mitochondria [141]. Ultimately, these processes lead not only to decreases of the PMF but also to reduction of the efficiency of oxidative phosphorylation and decreased generation of ROS [140].

**Figure 3:**
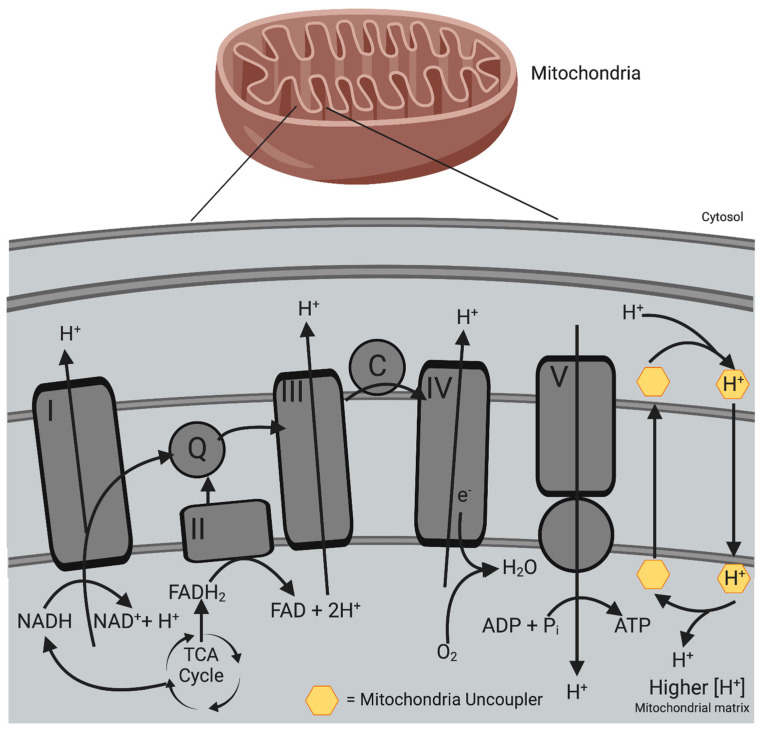
Protonophoric mitochondrial uncoupling mechanism. Mitochondrial uncoupling occurs when protons re−enter the mitochondrial matrix by bypassing ATP synthase (Complex V) without producing ATP. Protonophoric uncouplers aid the transport of protons (H^+^) across the inner mitochondrial membrane, dissipating the PMF. These protonophores bind to H^+^ in the intermembrane space, translocating them into the mitochondrial matrix. When protonophores are deprotonated, they can diffuse across the inner mitochondrial membrane and repeat the process. A decrease in the PMF leads to increased energy expenditure as the mitochondria need to increase nutrient oxidation in order to maintain ATP synthesis. Created with BioRender.com (accessed on 7 June 2023).

Synthetic mitochondrial uncouplers can act as protonophores whereby they transport protons from the intermembrane space across the inner mitochondrial membrane to the matrix in a protein-independent uncoupling process (Figure 3). This dissipates the PMF that is required for the coupling process between the ETC and ATP synthesis in oxidative phosphorylation [143].

The most well-known and studied mitochondrial uncoupler is 2,4 DNP, a protonophore whose mechanism of action is to dissipate the PMF, making ATP generation less efficient and leading to heat generation [144]. However, its lack of mitochondrial specificity and narrow therapeutic index led to adverse effects such as hyperthermia, cataracts, and hepatotoxicity, thereby causing several deaths and ultimately being banned by the FDA in 1938 [145,146,147,148,149]. Despite this, research on this compound has continued to identify its therapeutic applications. It was discovered that administering low doses of 2,4 DNP, which induced mild mitochondrial uncoupling, effectively delayed the progression of experimental atherosclerosis in hypercholesterolemic LDL-deficient mice [121]. The study revealed a reduction of 36% in lipid-stained areas of atherosclerotic lesions in the aortic root of LDL-deficient mice treated with 2,4 DNP. Additionally, in LDL-deficient mice fed an atherogenic diet, the reduction was 26% smaller in the 2,4 DNP-treated group [121]. While it did not reduce classical atherosclerosis markers such as plasma lipids and systemic inflammatory markers, it was shown to reduce superoxide production as well as systemic and tissue oxidative damage markers [121]. In addition, 2,4 DNP has been connected to a lipophilic triphenylphosphonium cation (TPP), a mitochondrial delivery vector, by a photolabile linker which releases 2,4 DNP into the mitochondria upon irradiation in order to overcome its lack of mitochondrial specificity [150]. 

Nitazoxanide is another compound that has been repurposed for its mitochondrial uncoupling effects. It is an FDA-approved oral antiparasitic and antiviral drug [144]. A recent study published data indicating its ability to inhibit the formation of atherosclerotic plaques in atherosclerotic mice fed a western diet [115]. The success of existing mitochondrial uncouplers like DNP and nitazoxanide has sparked a resurgence in the development of second-generation mitochondrial uncoupler compounds, such as BAM15, which exhibits highly specific activity in the mitochondria and low cytotoxicity [116]. Moreover, BAM15 has been shown to significantly increase mitochondrial OCR even in the presence of the ATP synthase inhibitor oligomycin. It also effectively stimulates mitochondrial OCR in mitochondria respiring on pyruvate, malate (Complex I substrates), or succinate (Complex II substrate) [116]. In normal murine liver (NMuLi) cells, BAM15 was approximately 7 times more potent than 2,4 DNP at stimulating oxygen consumption rate and sustaining high levels of mitochondrial respiration across a broad concentration range (3–100 μM) [151]. Thus, mitochondrial uncouplers hold significant value in the treatment of metabolic diseases and disorders, including atherosclerosis.

### 4.2. ROS Scavengers

ROS scavengers are designed, as per its namesake, to scavenge and deplete ‘detrimental’ ROS which subsequently can delay or inhibit oxidative stress in ROS-rich microenvironments [125,152]. Like mitochondrial uncouplers, ROS scavengers can be divided into either naturally occurring or synthetic ROS scavenging agents [152]. The human body possesses enzymatic and non-enzymatic free radical scavenging systems. The enzymatic free radical scavenging systems are a group called metalloenzymes which include superoxide dismutase (SOD), glutathione catalase (GSH), and catalase (CAT) which protect against ROS through the dismutation of free radicals such as O_2_^−^ and H_2_O_2_ into O_2_ and H_2_O [153]. Non-enzymatic ROS scavenging systems include vitamin A, vitamin C, and vitamin E, which prevent or protect against oxidation or oxidative damage [154]. However, most natural ROS scavengers are small molecules that have inherent instability, weak ROS scavenging ability, and short circulating half-life that cannot protect against the overproduction of ROS [155]. Therefore, synthetic ROS scavenging agents with strong ROS scavenging ability and a long half-life are a viable therapeutic strategy against oxidative stress caused by chronic inflammatory diseases like atherosclerosis. One of the first ROS scavengers developed was mitovitamin-E (MitoVit-E). It sought to utilize the antioxidant property of vitamin E, covalently attach it to TPP, and deliver it specifically to the mitochondria [156]. MitoVit-E saw positive results, having been shown to decrease ROS production and apoptosis in bovine aortic endothelial cells (BAECs) exposed to oxidative stress [124]. However, Vitamin E is not a catalytic antioxidant and its non-regenerative scavenging activity makes it a temporary solution to chronic, inflammatory conditions like atherosclerosis [125]. 

Similarly, MitoQ is a synthetic coenzyme Q_10_ analogue that is bound covalently to TPP which allows it to have high specificity at the mitochondria [56,157]. Coenzyme Q_10_ (CoQ_10_) is present in all respiring eukaryotic cells as a member of the electron transport chain [157]. It functions as an electron carrier in the mitochondrial inner membrane where it assists in the transport of e^−^ from Complex I to Complex II and from Complex I to Complex III [158]. CoQ_10_ also transfers H^+^ to the mitochondrial intermembrane space [159]. This transfer of e^−^ and H^+^ creates a proton gradient which drives the production of ATP [160]. CoQ_10_ in its reduced form, ubiquinol (CoQ_10_H_2_), has antioxidant scavenging activity in order to protect against lipid peroxidation by regenerating tocopherol, an antioxidant, which prevents cell damage caused by oxidative stress [161]. MitoQ is also able to effectively protect against mitochondrial lipid peroxidation and peroxynitrite-induced damage due to its sub-localization to the matrix-facing side of the inner mitochondrial membrane where it can be recycled by Complex II into ubiquinol [162]. In this form, it can donate a hydrogen atom to oxygen-centered radicals, thereby neutralizing it and inhibiting oxidative damage to mitochondrial lipids, proteins, and DNA [162]. However, unlike MitoVit-E, MitoQ has regenerative scavenging activity whereby after donating its hydrogen atom to an oxygen-centred radical, it becomes ubisemiquinone, which quickly dismutates back into CoQ_10_ and CoQ_10_H_2_. CoQ_10_ can then be recycled into CoQ_10_H_2_ by Complex II to participate in the process again [163]. 

In vitro, MitoQ has been shown to protect against oxidative stress induced by cigarette smoke extract (CSE)-treated HUVECs where it was able to alleviate ROS levels in CSE-treated HUVECs by approximately 50% (*p* < 0.01) [126]. Contrary to expectations, a study investigating the bioenergetic effects of MitoQ discovered that the compound possesses pro-oxidant properties [164]. The study revealed that MitoQ exhibited a significant reduction in OCR_ATP_ (150–300 nM for 18 h) in a dose-dependent manner while simultaneously increasing mROS and H_2_O_2_ production in bovine aorta endothelial cells (BAECs) [164]. In vivo, MitoQ was able to restore the age-related decline in NO bioavailability and intracellular and mitochondrial superoxide production, all hallmarks of endothelial dysfunction, in aged C57/BL6 mice [127]. In addition, MitoQ has been shown to improve mitochondrial dysfunction in rats with heart failure induced by pressure overload by decreasing H_2_O_2_ production and restoring mitochondrial respiration in state 3 conditions or maximal oxygen consumption rate (OCR) (by addition of 200 μM ADP) and improving mPTP opening [128]. This suggests its potential in treating atherosclerosis and CAD, whereby its regenerative scavenging activity and ability to resolve endothelial dysfunction make it highly promising. 

Another antioxidant and ROS scavenger is NAC. NAC is an FDA-approved drug and is recognized by the World Health Organisation (WHO) as an essential drug for the treatment of paracetamol overdose [165]. It is an acetylated cysteine residue that acts as a precursor for GSH synthesis [166]. The direct antioxidant activity of NAC is through the reaction of its free thiol group with ROS and reactive nitrogen species (RNS) [167]. Indirectly, it restores GSH levels and increases SOD activity which also aids in reducing oxidative stress and ROS production [168,169,170]. NAC has been shown to reduce atheroma progression in uremic-enhanced apolipoprotein E knockout mice [129]. Moreso, it has been shown to have mitochondria-specific activity; however, its mechanism of action is debated. Ali et al. proposed that NAC protects against mitochondrial dysfunction in the brain of ischemia/reperfusion injury rat models via the regulation of p-GSK-3β-mediated Dynamin-related protein 1 (Drp-1) translocation to the mitochondria [171]. Another study theorised that NAC replenishes mitochondria GSH, which subsequently protects against oxidative stress and cell death [172]. In human hepatoma (HepG_2_) cells treated with 10 μM sodium selenite, mitochondrial membrane potential (MMP) depolarisation was reduced by 50% as well as significantly inhibiting cytochrome *c* release [173]. However, its effects remain to be seen in endothelial cells. Nonetheless, NAC can be seen as a highly promising compound of interest in preventing the onset of atherosclerosis through attenuating mitochondrial dysfunction in atherosclerotic endothelium. 

Recently, research has been conducted on the formulation of ROS-scavenging biopolymers [152]. This includes the development and use of biopolymers such as nanoparticles, micelles, microspheres, and hydrogels for anti-inflammatory applications [152]. So far, lipid-rich micelles nanoparticles combined with loading andrographolide have been shown to not only act as a responsive and stimulating drug carrier to release encapsulated compounds but also demonstrate the ability to consume ROS at pathological sites in atherosclerosis [130]. This poses a fascinating possibility of providing immediate relief at inflammatory sites but also through the release of encapsulated compounds such as mitochondrial uncouplers or ROS scavengers for subsequent relief against atherosclerosis.

### 4.3. P2X7R Antagonists 

P2X7R antagonists are compounds that target and block the activity of P2X7R. P2X7R antagonists have been developed as potential therapeutic agents for the treatment of various diseases associated with P2X7R activation which include inflammatory disorders and cardiovascular disease [112,174,175,176]. P2X7R antagonists have been shown to have anti-inflammatory effects and have been investigated as potential therapeutic agents for the treatment of inflammatory disorders such as rheumatoid arthritis and inflammatory bowel disease [174,175]. In the context of cardiovascular disease, P2X7R antagonists have been shown to have vasodilatory effects and have been investigated as potential therapeutic targets for the treatment of diseases such as hypertension and atherosclerosis [112,176]. 

P2X7R antagonists are divided into two groups: non-selective ATP-derivative antagonists and non-ATP antagonists [133]. There are two non-selective ATP-derivative antagonists, TNP-ATP and periodate-oxidised ATP (oATP), that have high μmol potencies which make them less optimal as a form of treatment [177,178]. As a result, the majority of developed P2X7R antagonists are non-ATP-based compounds. The mechanism of action of P2X7R antagonists is dependent on their interaction with the receptor [133]. Some behave as orthostatic antagonists which competitively bind to the ATP binding pocket; however, the majority are allosteric antagonists that reduce the binding affinity of ATP to the P2X7R by binding to locations other than the ATP binding site [133,179]. 

A novel P2X7R antagonist with therapeutic potential is PKT100/SMW139. It is a second-generation antagonist that has been previously demonstrated to inhibit the inflammasome in the PBMCs of STEMI patients, leading to a significant reduction in caspase-1 and IL-1β activity by 60% and 13.9%, respectively [131]. In vivo, PKT100 was found to improve the cardiac function and survival of C57BL/6 mice with pulmonary fibrosis and secondary pulmonary hypertension [132]. Mice treated with bleomycin and PKT100 showed approximately 40% less RV dysfunction as well as a 20% improvement in right ventricular systolic excursion velocity when compared to the bleomycin-vehicle control cohort [132]. Mice treated with bleomycin and PKT100 also displayed a 36% increase in survival compared to the bleomycin-vehicle control group [132]. In addition, Hansen et al. found improvements in inflammation between DMSO control and bleomycin-treated C57BL/6 mice, whereby IL-1β was significantly reduced to the levels of the sham mice [132]. Overall, this showcases its potential for treating atherosclerosis by resolving endothelial and mitochondrial dysfunction. 

Another P2X7R antagonist of interest is AZD9056. It was developed for the treatment of inflammatory diseases such as RA and CD [134,135]. It has previously entered clinical trials for the treatment of patients with RA and CD; however, it was found to not have significant efficacy in phase II [133,134,135]. Despite disappointing results and not being a therapeutically useful target for these inflammatory diseases, it may have more promising results in treating other inflammatory diseases such as atherosclerosis that make it worth investigating. 

### 4.4. Colchicine

Colchicine is an anti-inflammatory agent of prospective therapeutic use. Its mechanism of action is by binding tubulins, which causes microtubule depolymerisation and thereby affects a variety of cellular processes including the maintenance of cell shape, signalling, and transport [180,181]. Colchicine interferes with several inflammatory processes including superoxide production and inflammasome activation. A study involving HUVECs treated with cholesterol crystals to induce endothelial cell inflammation and pyroptosis revealed that colchicine alleviates cellular oxidative stress and NLRP3 inflammasome activation by regulating the AMPK/SIRT1 signalling pathway [182]. Interestingly, colchicine’s effect on inflammasome-linked pathologies has also been linked to down-regulating the P2X7 receptor [183]. The study demonstrated that colchicine potently inhibited the pore formation of P2X7 receptors in vitro and in vivo [183]. The formation of pores is a critical step in triggering ATP-induced NLPR3 inflammasome activation [184]. Colchicine treatment in peritoneal mouse macrophages and mice inoculated with lipopolysaccharide (LPS) and ATP had diminished ROS production [183]. 

As a potent anti-inflammatory compound, colchicine has been proposed to have cardioprotective properties in patients with atherosclerosis [136,137]. The low-dose colchicine for secondary prevention of cardiovascular disease (LoDoCo) clinical trial was used to address the efficacy of colchicine in atherosclerosis and showed that 0.5 mg/day treatment significantly decreased the risk of the primary combined endpoint (combination of cardiovascular death, ischaemic stroke, spontaneous myocardial infarction, and coronary re-vascularisation caused by ischaemia) by 4.6–16% [136]. A follow-up study, LoDoCo2, was completed in 2020 with a larger sample size and the same primary endpoint, and it also confirmed that a low dose of colchicine (0.5 mg/day) decreased the incidence of CVD by 6.8–9.6% [137]. 

### 4.5. MPO Inhibitors

MPO inhibition operates under one of three general mechanisms. The first one involves the heme centre of the enzyme, which is where HOCl is formed [185]. Irreversible inhibitors can form strong, covalent bonds with the iron atom of the heme center with blocks H_2_O_2_ from accessing the active site, inactivating MPO [185]. The second mechanism involves competitive inhibition between the MPO inhibitor and the enzyme-substrate for the active site. The reversible inhibitor forms the complex with MPO in order to prevent the MPO peroxidase cycle from continuing [185]. Alternatively, HOCl could be scavenged which can prevent oxidative and tissue damage. However, the peroxidation cycle and formation of ROS will continue [185]. Inhibition of MPO has been demonstrated to reduce endothelial dysfunction in mouse models of vascular inflammation and atherosclerosis [115]. This study utilized the compound AZM198 to treat three different models of inflammation: femoral cuff, tandem stenosis in ApoE^−/−^ mice, and insulin resistance in C57BL/6J mice fed a high-fat, high-carbohydrate diet [115]. They found that endothelial function improved in all models except for the insulin resistance model, and this may be because of the inhibition of MPO’s chlorinating activity, which was significantly reduced in these models [115].

## 5. Future Directions 

In this review, we have identified mitochondrial dysfunction in endothelial cells as a potential biomarker of atherosclerotic disease. Although a wide variety of targets, compounds, and technology are available to treat atherosclerosis at its earliest stage, we are still far from curing it. Humans are innately unique given that plaque development in the coronary arteries is absent in other mammalian species [186]. In particular, our closest evolutionary relatives, chimpanzees share ~96% similarity with our genome, yet only a single case of myocardial infarction has been reported with evidence in right coronary artery plaque rupture [186,187]. This is despite having highly atherogenic lipid profiles with higher levels of LDL cholesterol (~4.4 mmol/L vs. ~2.8 mmol/L) and lipoprotein (a) (0.61 mg/mL vs. 0.18 mg/mL) than the average human [188,189,190,191]. Given the similarities in our genomic profiles, evaluation of these differences could elucidate potential mechanisms and drug targets for the treatment of CAD. 

Current preclinical models used for the evaluation of lesion development are conducted in rodents that experience a vastly different pathology of atherosclerosis compared to humans [192,193,194]. In particular, the coronary arteries are spared in mice with other parts of the arterial tree affected such as the aortic root and arch and brachiocephalic trunk, whereas in humans, the coronary arteries, carotid, and peripheries are mostly impacted [192,193,194]. This proposes the need to move to more ‘humanized’ models for atherosclerosis research.

### 5.1. Endothelial Colony Forming Cells and Importance in Biomarker and Drug Discovery

First identified in 1997 by Asahara et al., endothelial progenitor cells (EPCs) are circulating cells that possess endothelial surface markers and repair ability and that share the phenotypic characteristics of the vascular endothelium [195,196]. Over the years they have been redefined leading to two categories: early outgrowth EPCs and late outgrowth EPCs. Early outgrowth EPCs have haemopoietic origins that promote angiogenesis through paracrine signalling; however, they are unable to form mature endothelial cells [197]. 

On the other hand, late outgrowth EPCs or endothelial colony-forming cells (ECFCs) are a rare progenitor cell population derived from PBMCs that possess high proliferative capacity, express endothelial cell markers, and behave similarly to mature endothelial cells both phenotypically and functionally [195,198,199]. The origin of circulating ECFCs is still unclear. Earlier studies suggested that they were derived from bone marrow; however, more recent studies suggest a tissue vascular or microvascular niche background [196,200,201]. Recently, it has been shown by Besnier et al. and Boer et al. that patient-derived ECFCs reflect the patient’s diseased or healthy endothelial function, both of which highlight their use as in vitro and ex vivo models to study vascular disease, respectively [202,203]. Besnier et al. demonstrated a positive correlation between mROS production and a patient’s CAD severity [202]. These studies show how it is possible to characterise phenotypic and molecular dysfunctions in diseases such as CAD with heterogenous clinical presentations. ECFCs have been used previously for cell and gene therapy and tissue bioengineering. The review by Paschalaki and Randi goes through the aforementioned in depth [197]. 

However, the application of ECFCs in drug discovery has not been investigated. Their retention of individual patient characteristics is critical for the understanding of a drug or compound’s efficacy in patient-to-patient variability. Much like how clinical trials observe a compound’s effects in real patients, the use of ECFCs allows for this variability to be observed ex vivo. Taking it a step further, ECFCs would allow for high-throughput compound screenings to take place for a variety of diseases that patient-derived ECFCs may reflect, allowing for the rapid identification of key molecules. 

Despite the promising nature of ECFCs, they are not without drawbacks. Notably, the formation of colonies from PBMCs takes anywhere from 10 to 21 days (on average 13.7 days as well as the low success of spontaneous growth; 21.5%), making it sub-optimal for use as an immediate, translatable diagnostic or prognostic tool for patients [202]. Rather, reverse-engineering a biomarker present in ECFCs that is also found in its precursor, PBMCs would be more useful and highly significant for patients at-risk of developing CAD. 

### 5.2. Phenotypic High Throughput Screening for Treating Mitochondrial Dysfunction in Endothelial Cells 

High-throughput screening (HTS) is essential in the drug discovery process, and its implementation has been key in the identification and validation of novel drugs [204,205,206]. Traditionally, HTS has been conducted from a compound library to determine a compound’s ability to inhibit the enzymatic activity or binding property of a purified target protein [207]. This is typically achieved using a microplate reader to perform readouts. Phenotypic HTS utilises the phenotypes present within cells, tissues, or organisms as a key component for discovering compound efficacy [208]. Such a technique has been applied previously in atherosclerotic research to identify compounds that protect against the development of atherosclerosis [209,210,211]. 

As discussed previously, a dysfunctional endothelium is an early indicator of atherosclerosis [41]. However, there is a lack of research performed to target the endothelium despite its importance and association with the disease. In the context of atherosclerotic research and phenotypic HTS, macrophages and monocytes are the cell line of choice that are investigated. This is because they play an important role in the progression of atherosclerosis in which, at atherosclerotic lesions, they participate in the ingestion and accumulation of lipoprotein, which creates foam cells. The accumulation of foam cells contributes to atherosclerotic plaque growth [212]. Nonetheless, combining patient-derived ECFCs that retain disease phenotypes with drug screening would be useful in identifying key compounds useful in not only treating mitochondrial dysfunction within endothelial cells but in identifying potential inflammatory and oxidative pathways. 

### 5.3. Omic Approaches to Drug and Biomarker Discovery with ECFCs

Currently, drug discovery is limited to existing and known biomarkers. For example, parameters to measure mitochondria health include the generation of mitochondria superoxide, and membrane potential through dyes such as MitoSOX and MitoTracker Deep Red, respectively. However, there are no biomarkers that directly examine the mitochondria in a high-throughput manner. The application of omics-based technologies can solve this issue. We can combine different omics approaches such as proteomics, metabolomics, and transcriptomics and examine all parts of/associated with the mitochondria to detect differences between patient cohorts with and without coronary artery disease. From there, biomarkers can be identified through multi-omic analysis and drugs can be specifically developed to target it directly. This was performed recently in which a multi-omic screen identified molecular biomarkers that were causally associated with the risk of CAD [213]. Alternatively, one could conduct phenotypic HTS of compounds to identify potential candidates followed by omics analysis to investigate how these compounds impact known cellular/protein changes and uncover biomarkers using this approach. Regardless, the power of omics allows for major drug and biomarker discoveries to be made possible. 

### 5.4. Animal Models of Atherosclerosis to Study Endothelial Mitochondrial Dysfunction 

Animal models used to study atherosclerosis are highly informative in the investigation of its pathogenesis, mechanisms, and complications. Predominantly, mice are used as the species of choice for atherosclerotic studies, mainly due to their small size, relatively low cost, and ease of genetic modification [192,214,215]. Two models used to study atherosclerosis are dietary and/or genetic models. A variety of atherogenic dietary murine models, which involve a high-fat diet with various concentrations of cholesterol, have been used to induce major lesion development. A high-fat diet facilitates the accumulation of larger very-low-density lipoprotein (VLDL) and remnant lipoproteins (RLP) as well as elevated plasma cholesterol levels [192]. 

In terms of genetically modified mouse models, two common ones used in murine atherosclerosis are apolipoprotein E knockout (ApoE)^−/−^ and the low-density lipoprotein receptor (LDLR)^−/−^ models. The ApoE knockout model was the first genetically modified atherogenic murine model developed in which ApoE knockout mice exhibited significant hypercholesterolemia, having a five-fold increase in plasma cholesterol level relative to wild-type mice despite being on a low-fat diet [216,217,218,219]. ApoE is a lipoprotein constituent except for LDL and is an important ligand for LDLR-mediated removal of lipoprotein remnants from circulation [215,217]. The other model developed is the LDLR-deficient murine model. LDLR has fewer functions when compared to ApoE, and the absence of LDLR can be more easily attributed to lipoprotein homeostasis than other cellular processes such as inflammation and proliferation [192]. A deficiency in LDLR leads to a greater prevalence of LDL as the plasma lipoprotein carrying cholesterol because of an impaired ability to uptake and clear lipoprotein [192]. These genetic models are sometimes combined with dietary models (e.g., western diet, high-fat diet) in order to exacerbate the atherosclerotic phenotype [220]. The advantages and disadvantages are highlighted in Table 2.

Animal studies involving mitochondrial dysfunction in atherosclerotic models have recently become a subject of interest. The relationship between mtDNA damage and oxidative stress has been investigated in detail. One study which involved double knockout of ApoE and mitochondrial DNA polymerase G (POLG) showed an accumulation of mtDNA damage in circulating cells and vessel walls, promoting atherosclerosis as well as being associated with the formation of vulnerable plaques [88]. 

Separately, endothelial dysfunction has also been investigated in atherosclerotic animal models. Endothelial dysfunction of ApoE^−/−^ rats fed a western diet was reported, which was associated with early-stage atherosclerosis and elevated LDL and triglyceride levels [231]. Interestingly, only one study has investigated both endothelial and mitochondrial dysfunction in an animal model of atherosclerosis. The study focused on the effects of endothelial cell-specific transgenesis of mitochondrial thioredoxin (Trx2 TG) in ApoE^−/−^ mice and its impact on atherosclerotic lesion formation. The researchers demonstrated the crucial role of Trx2 expression in endothelial cells in preserving endothelial function by enhancing NO bioavailability through scavenging mROS [232]. In isolated mouse endothelial cells (MECs), Trx2 TG MECs exhibited lower mROS levels compared to wild-type MECs under basal or stimulated conditions induced by TNF, paraquat, or H_2_O_2_ [232]. Furthermore, the expression of Trx2 in the endothelium of Trx2 TG/ApoE^−/−^ mice effectively prevented hypercholesterolemia-induced endothelial dysfunction by preserving NO levels and restoring vascular reactivity [232]. The study also revealed that Trx2 expression reduced hypercholesterolemia-induced atherosclerosis by reducing ROS levels and improving NO bioavailability in Trx2 TG/ApoE^−/−^ mice, resulting in significantly reduced lesions compared to ApoE^−/−^ mice [232]. Considering this, other studies that could be performed include testing potential compounds that could halt or prevent long-term atherosclerosis development in these models of atherosclerosis that specifically investigate endothelial mitochondrial dysfunction. 

## 6. Conclusions

Dysregulated redox signalling plays a key role in the development of chronic diseases like CAD. As such, gaining insights into the degree of redox dysregulation at the cellular level is paramount. Innovations in molecular biology have allowed us to identify several novel targets and compound classes that could be used in treating the disease in endothelial cells. Using high-throughput methods such as HTS and omics (transcriptomics to proteomics), we can combine them with patient-derived models of the endothelium (ECFCs) in order to unravel novel biological mechanisms which contribute heavily to atherosclerotic development. However, the exact composition of the mitochondria that primarily causes dysregulation is yet to be discerned. 

## Figures and Tables

**Table 1:** Compounds and their therapeutic potential for treating mitochondrial dysfunction.

Compound	Mechanism of Action	Mitochondria Specificity?	Used in Cardiovascular Setting?	Clinical Trials?	References
2,4 dinitrophenol (2,4 DNP)	Mitochondrial uncoupling	No	No	No	[121]
Nitazoxanide	Mitochondrial uncoupling	No	Yes. Shown to reduce formation of atherosclerotic plaques in atherosclerosis-model mice fed western diet.	No	[122]
BAM15	Mitochondrial uncoupling	Yes	No	No	[123]
MitoVit-E	ROS scavenging	Yes	No	No	[124,125]
Mitoquinone (MitoQ)	ROS scavenging	Yes	Yes. Shown to reduce hydrogen peroxide production, improve mitochondrial respiration and mitochondrial permeability transition pore (mPTP) opening.	Yes. None for CAD.	[126,127,128]
N-acetylcysteine (NAC)	ROS scavenging	No	Yes. Shown to reduce atheroma progression in uremic-enhanced apolipoprotein E knockout mice.	Yes. None for CAD.	[129]
ROS-scavenging biopolymers	ROS scavenging	No	Yes. Micelles combined with loading andrographolide have been shown to act as a responsive and stimulating drug carrier to release encapsulated compounds and demonstrate the ability to consume ROS at pathological sites.	No	[130]
PKT100	P2X7R antagonists	No	Yes. Human peripheral blood mononuclear cells (PBMCs) derived from STEMI patients have inhibited inflammasome. Mice with pulmonary fibrosis and hypertension have improved cardiac function and survival. Bleomycin-treated mice have reduced right-ventricular (RV) dysfunction, improved right ventricular systolic excursion velocity, survival and reduced IL-1β.	No	[131,132]
AZD9056	P2X7R antagonists	No	No	Yes. None for CAD.	[133,134,135]
Colchicine	Anti-inflammatory, non-specific	No	Yes. Low dose colchicine (0.5 mg/day) significantly decreased risk of cardiovascular adverse events.	Yes, LoDoCo (pilot study) and LoDoCo2 (randomized follow-up trial). However, incidence of death from non-cardiovascular disease was higher in the colchicine group	[136,137]
AZM198	MPO Inhibitor	No	Yes. Shown to reduce endothelial dysfunction in mouse models of vascular inflammation and atherosclerosis.	No	[115]

**Table 2:** Advantages and disadvantages of atherosclerotic genetic murine models.

Genetic Model	Advantages	Disadvantages
ApoE^−/−^	This model demonstrates a higher degree of similarity in the development of atherosclerosis compared to humans [221]. Rapid lesion development [222,223].	Lipid metabolism differs from humans with majority of plasma cholesterol is VLDL not LDL [224,225]. Exhibits additional atheroprotective properties alongside its role in regulating lipoprotein clearance [226]. No significant coronary artery lesion manifestation [192].
LDLR^−/−^	This model shares characteristics observed in human familial hypercholesterolemia, which is more commonly associated with the absence of functional LDLR rather than apoE deficiency [227,228]. The absence of LDLR is advantageous for attributing lipoprotein homeostasis, as there are fewer associated functions of LDLR that may influence the interpretation [192].	Older LDLR-deficient mice on a standard diet exhibit limited development of lesions [229,230]. No significant coronary artery lesion manifestation [192].
